# Emerging complexities of phospholipid metabolism in adipose tissue

**DOI:** 10.1016/j.mocell.2026.100333

**Published:** 2026-02-23

**Authors:** Minju Kim, Hyunju Lee, Joerg Heeren, Junho Kim, Gakyung Lee, Su Myung Jung

**Affiliations:** 1 Department of Biological Sciences, Sungkyunkwan University (SKKU), Suwon, South Korea; 2 Department of Biochemistry and Molecular Cell Biology, University Medical Center Hamburg-Eppendorf, Hamburg, Germany; 3 Department of Integrative Biological Sciences and Industry, Sejong University, Seoul, South Korea

**Keywords:** Adipose tissue, Adipocyte, Phospholipid, Lands’ cycle, Lysophospholipid

## Abstract

Adipose tissue centrally regulates lipid homeostasis through its robust metabolic flexibility in response to nutritional states and environmental cues. Adipose lipid metabolism has been studied primarily from a neutral lipid-centric perspective; however, advances in lipid research technologies are beginning to uncover the critical roles of polar lipids in adipose tissue function. Here, we review emerging roles of phospholipid metabolism, particularly phospholipid biosynthesis, remodeling through the Lands' cycle, and lysophospholipid signaling in adipocytes. Understanding the complexities of phospholipid metabolism in adipose tissue opens new avenues for therapeutic interventions targeting obesity and metabolic disorders.

## Graphical abstract

**Figure:**
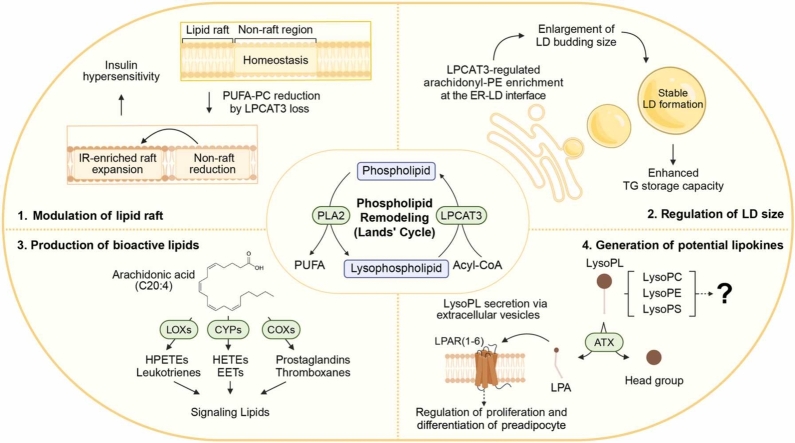

## INTRODUCTION

Obesity, defined as excessive accumulation of adipose tissue, is a central risk factor for metabolic disease and shows strong associations with the development of type 2 diabetes and cardiovascular disease (Donohoe et al., 2017, Flegal et al., 2013). Despite its clinical importance, the metabolic and physiological understanding of adipose tissue has a relatively short research history compared with other metabolic organs such as the liver and skeletal muscle, though it has gained considerable attention in recent decades. Growing evidence suggests that pathophysiological alterations in adipose tissue play crucial roles in the development of metabolic disease, leading to active efforts to establish therapeutic strategies for metabolic disorders through a deeper understanding of adipose tissue biology (Sakers et al., 2022).

Adipose tissue largely consists of 2 types of adipocytes with respect to lipid handling. Conventional adipocytes (called white adipocytes) are mainly located in subcutaneous and visceral adipose tissue depots (SAT and VAT, respectively) and primarily function as energy storage sites and key endocrine organs (Kim and Moustaid-Moussa, 2000, Trayhurn and Beattie, 2001, Vazquez-Vela et al., 2008). They efficiently take up circulating lipids, converting them into free fatty acids (FFAs) that are then re-esterified into triglycerides (TGs) and stored within a single large lipid droplet (Sakers et al., 2022). During periods of energy deficit, such as fasting, these stored TGs are broken down through a process called lipolysis, releasing FFAs back into circulation to be used as an energy source (Arner, 2005, Fruhbeck et al., 2014, Grabner et al., 2021). In contrast, thermogenic adipocytes, largely consisting of brown adipocytes in brown adipose tissue (BAT) and beige/brite (brown-in-white) adipocytes in SAT, are functionally and morphologically distinct, characterized by multilocular lipid droplets and high mitochondrial content (Cannon and Nedergaard, 2004, Sidossis and Kajimura, 2015).

Thermogenic adipocytes are specialized to dissipate energy from both intrinsic and extrinsic lipids through nonshivering thermogenesis, a process primarily mediated by uncoupling protein 1 (UCP1). FFAs derived from intracellular lipolysis and circulating TG-rich lipoproteins are transported to mitochondria for fatty acid oxidation (Bartelt et al., 2011, Cannon and Nedergaard, 2004, Grabner et al., 2021). In addition to fatty acid oxidation, de novo lipogenesis (DNL) is paradoxically upregulated in BAT during chronic cold adaptation; this process is essential for optimal thermogenesis, at least in part by mitigating mitochondrial stress (Korobkina et al., 2024, Sanchez-Gurmaches et al., 2018). Conversely, inhibiting lipogenesis prevents the metabolic inactivation of BAT during thermoneutral adaptation (Schlein et al., 2021). Recent single-nucleus transcriptomics studies further reveal that BAT harbors a distinct population of lipogenic adipocytes; notably, this subpopulation is transiently suppressed during acute cold exposure but rebounds significantly afterward (Behrens et al., 2025, Lundgren et al., 2023). Furthermore, inhibiting DNL in mature adipocytes has been shown to promote thermogenesis via SAT browning (Guilherme et al., 2023, Lodhi et al., 2012). Collectively, these findings suggest that the precise regulatory roles of DNL in the thermogenic program require further investigation.

In addition to lipids, thermogenic adipocytes utilize diverse substrates to meet their high metabolic demands. Cold exposure significantly increases glucose uptake, allowing both glycolytic and mitochondrial oxidation to contribute to heat production and biosynthesis, with the latter becoming prominent during chronic cold adaptation (Jung et al., 2021, Wang et al., 2020). Furthermore, recent metabolomics and isotope tracing studies show cold-activated BAT recruits a broad range of amino acids; for instance, branched-chain amino acids and glutamine are catabolized to fuel the tricarboxylic acid cycle (Lee et al., 2023, Park et al., 2023, Yoneshiro et al., 2019). During mitochondrial oxidation, the electron transport chain generates a proton gradient, but UCP1 facilitates a proton leak across the inner mitochondrial membrane, dissipating energy as heat rather than ATP (Cannon and Nedergaard, 2004, Cero et al., 2021, Gonzalez-Hurtado et al., 2018). Importantly, recent findings indicate that UCP1-independent mechanisms, such as futile metabolic cycles, also contribute to heat generation (Roesler and Kazak, 2020, Sharma et al., 2024).

Historically, research on adipose lipid metabolism has primarily focused on neutral lipids, particularly TGs. These have been extensively studied using classical biochemical approaches to understand their biosynthesis and breakdown, providing fundamental insights into energy storage and mobilization in adipose tissue (Ahmadian et al., 2007, Brasaemle, 2007, Brasaemle et al., 2004, Haemmerle et al., 2006, Lass et al., 2006, Osuga et al., 2000, Tansey et al., 2001, Zimmermann et al., 2004). However, recent advances in high-throughput lipidomic profiling have expanded the field, enabling the systematic characterization of diverse lipid classes and shifting scientific attention beyond TGs (Cho et al., 2023, Cho et al., 2022, Huynh et al., 2019, Lange et al., 2021, Leiria et al., 2019, Lynes et al., 2018, Marcher et al., 2015, May et al., 2017, Pietilainen et al., 2011, Sato et al., 2020, Simcox et al., 2017, Tol et al., 2025, Yore et al., 2014). Among these, phospholipid metabolism—long underappreciated despite its critical roles in membrane structure, lipid droplet dynamics, and cellular signaling—has only recently begun to be explored in detail (Table 1). This review will synthesize the emerging findings on phospholipid metabolism in adipose biology and highlight its importance in regulating adipose tissue function and systemic metabolic homeostasis.

**Table 1:** Phospholipid metabolic studies in adipose tissue using genetics, pharmacological, and supplementation approaches

Type of lipids	Targets and methods	Key findings	Key references
PC	**CCT**: RNAi	Increased CCT level during adipocyte differentiation **CCT depletion**: increasing LD size; decreasing LD number	Aitchison et al. (2015)
	**PEMT**: whole-body KO	Increased PEMT level during adipocyte differentiation **PEMT loss**: decreasing lipid anabolism and UCP1 expression; cold intolerance	Gao et al. (2015) Johnson et al. (2020)
PS	**PS**: supplementation	Increased plasma PS level after cold exposure **PS treatment**: increasing UCP1 level, with improved mitochondrial function and lipolysis	Zhou et al. (2024)
CL	**CRLS1**: OE, RNAi, and adipose tissue-specific KO	Increased CRLS1 level during cold adaptation **Ectopic CRLS1**: enhancing uncoupled respiration **CRLS1 loss**: resulting in mitochondrial dysfunction	Schlame and Greenberg (2017) Lee et al. (2015) Fromme et al. (2018) Sustarsic et al. (2018)
	**TAZ**: RNAi, whole-body KD	Increased TAZ level during adipocyte differentiation **TAZ loss**: disrupting mitochondrial structure and impairing thermogenesis	McKenzie et al. (2006) Phoon et al. (2012) Johnson et al. (2020)
LysoPL	**LPA**: supplementation	Elevated LPA level upon HFD **LPA treatment:** regulating preadipocyte proliferation and differentiation	Holmstrom et al. (2010) Li et al. (2011)
	**ATX**: whole-body KO, adipose tissue- specific KO, and inhibitor	Elevated ATX level upon HFD ATX modulates adiposity in context-dependent manner	Nishimura et al. (2014) Rancoule et al. (2013) Dusaulcy et al. (2011) D'Souza et al. (2018)
	**LysoPC**: supplementation	**LysoPC treatment:** facilitating glucose uptake and respiration; antiobesity effects	Yea et al. (2009b) Ma et al. (2021) Han et al. (2021)
PUFA-PL	**PLA2**: inhibitor (PLA2G2A), whole-body KO (PLA2G16 or PLA2G2D)	PLA2G16 is upregulated by feeding **PLA2G16 loss**: protecting against obesity via reducing eicosanoids **PLA2G2D loss**: impairing adipocyte browning and thermogenesis	Iyer et al. (2012) Jaworski et al. (2009) Sato et al. (2020)
	**LPCAT3**: adipose tissue-specific KO	Increased LPCAT3 in response to cold stress LPCAT3 regulates insulin sensitivity and LD size by modulating membrane physical properties	He et al. (2023) Tol et al. (2025) Holthuis and Menon (2014) Shimanaka et al. (2025)
Plasmalogen	**TMEM86A**: adipose tissue- specific KO	Increased TMEM86A level upon HFD **TMEM86A loss**: increasing LPE P-18:0, thus protecting against HFD-induced metabolic impairment	Cho et al. (2022)
	**LPE P-18:0**: supplementation	**LPE P-18:0 treatment**: protecting against DIO	Cho et al. (2022)
Ceramide	**SPTLC**: brown adipocyte-specific KO	The rate-limiting enzyme of ceramide biosynthesis **SPTLC loss**: protecting against DIO	Chaurasia et al. (2021)
	**ASAH1**: brown adipocyte-specific KO	The key enzyme of ceramide degradation **ASAH1 loss**: promoting DIO	Chaurasia et al. (2021)

ATX, autotaxin; CL, cardiolipin; CCT, cytidylyltransferase; LD, lipid droplet; LPA, lysophosphatidic acid; LPCAT3, lysophosphatidylcholine acyltransferase 3; PC, phosphatidylcholine; PEMT, phosphatidylethanolamine N-methyltransferase; PLA2, phospholipase A2; PS, phosphatidylserine; PUFA-PL, polyunsaturated fatty acids containing phospholipid; TAZ, tafazzin; UCP1, uncoupling protein 1.

## PHOSPHOLIPID BIOSYNTHESIS

Phospholipids are major components of cellular membranes, maintaining membrane fluidity, integrity, and functionality. Phospholipids consist of 1 hydrophilic phosphate-containing head group and 2 hydrophobic fatty acyl chains linked to glycerol backbone. The composition and distribution of phospholipids vary among different cellular compartments and organelles, contributing to their specialized functions (Spector and Yorek, 1985, van Meer et al., 2008, Wymann and Schneiter, 2008). Phospholipid biosynthesis primarily occurs in the endoplasmic reticulum (ER) through multiple pathways, most notably those responsible for the synthesis of phosphatidylcholine (PC) and its interconversion with phosphatidylethanolamine (PE) and phosphatidylserine (PS) (Vance, 2015). The detailed biochemistry of phospholipid biosynthesis pathways has been extensively reviewed elsewhere (Vance, 2015).

**Fig. 1:**
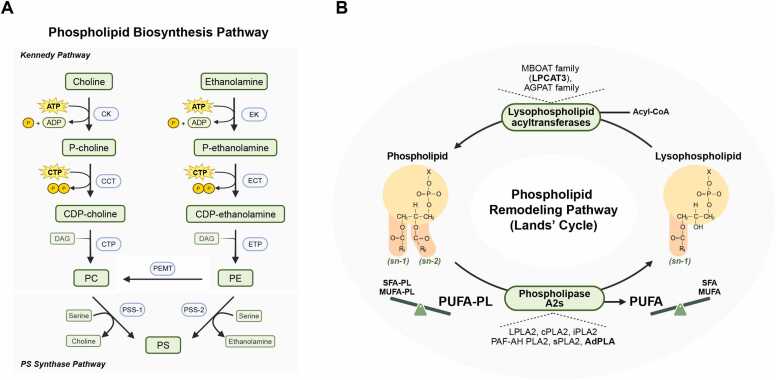
Phospholipid biosynthesis and remodeling pathways. (A) Phospholipid biosynthesis pathway. In Kennedy pathway, choline is first phosphorylated by choline kinase (CK) to produce phosphocholine, which is converted to CDP-choline by CTP:phosphocholine cytidylyltransferase (CCT). Subsequently, CDP-choline reacts with diacylglycerol (DAG) to form phosphatidylcholine (PC) via CDP-choline:1,2-diacylglycerol cholinephosphotransferase (CPT). Phosphatidylethanolamine (PE) can be synthesized through the CDP-ethanolamine pathway, a parallel branch of the Kennedy pathway. In mitochondria-associated membrane (MAM), PE can be methylated by phosphatidylethanolamine N-methyltransferase (PEMT) to yield PC. In PS synthase pathway, phosphatidylserine (PS) is synthesized by PS synthase-1 and -2 (PSS-1/2), which catalyze the exchange of head groups between PC or PE and serine. (B) Phospholipid remodeling pathway (Lands' cycle). The phospholipid remodeling pathway describes the continuous deacylation-reacylation process that remodels the acyl chains of phospholipids. Phospholipase A2s (PLA2s), which exist in indicated isoforms, hydrolyze the ester bond at the sn-2 position of phospholipids, generating lysophospholipids (LysoPLs) and releasing free fatty acids. The released fatty acids are predominantly polyunsaturated fatty acids (PUFAs). Lysophospholipids are subsequently reacylated by lysophospholipid acyltransferases (LPLATs), such as LPCAT3, which is the predominant isoform in adipose tissue.

In mammals, PC is mainly synthesized via the CDP-choline pathway, a branch of the Kennedy pathway (Fig. 1A) (Gibellini and Smith, 2010, Kennedy and Weiss, 1956). In this pathway, choline is first phosphorylated in the cytoplasm, and the resulting phosphocholine is then converted to CDP-choline by CTP:phosphocholine cytidylyltransferase (CCT), which is the rate-limiting enzyme under most physiological conditions (Fagone and Jackowski, 2013). CCT plays a pivotal role in lipid droplet expansion by synthesizing PC, which is essential for stabilizing the droplet surface and preventing coalescence. During lipid storage, CCT translocates to enlarging lipid droplet surfaces, where it becomes markedly activated to provide localized PC for the expanding phospholipid monolayer (Krahmer et al., 2011). This mechanism ensures that as the neutral lipid core grows, the surface is sufficiently coated with PC to maintain droplet integrity and prevent formation of abnormally large, dysfunctional droplets.

PE can be synthesized via the CDP-ethanolamine pathway, a parallel branch of the Kennedy pathway (Kennedy and Weiss, 1956). An alternative PC biosynthesis route involves PE N-methyltransferase (PEMT), which resides in the mitochondria-associated membrane and converts PE to PC (Fig. 1A) (DeLong et al., 1999, Li et al., 2006). Although this reaction occurs at substantial levels mainly in hepatocytes, studies suggest it may also be important in adipocytes, as CCTα and PEMT expression are reportedly upregulated during adipocyte differentiation in the 3T3-L1 cell culture model (Aitchison et al., 2015, Cole and Vance, 2010). Elevated expression of both CCT and PEMT has been correlated with adiposity in humans, underscoring the significance of PC biosynthesis in adipose tissue function (Sharma et al., 2013). However, in vivo studies using whole-body *Pemt* knockout mice show only marginal effects on anabolic lipid synthesis and no significant change in lipid catabolism (Gao et al., 2015). Other research has reported that systemic *Pemt* deficiency leads to impaired UCP1 expression and subsequent cold intolerance (Johnson et al., 2020). Nevertheless, whether these phenotypes reflect adipose tissue-intrinsic mechanisms remains unclear, particularly since BAT-specific deletion of *Pemt* does not alter UCP1 expression (Johnson et al., 2020). Further tissue-specific studies are needed to fully elucidate the role of PEMT in adipose tissue metabolism.

PS is biosynthesized primarily through base-exchange reactions, where the serine head group replaces the head group of either PC or PE. These reactions are catalyzed by PS synthases (PSS1 and PSS2), which ensure dynamic conversion among the major membrane phospholipids (Fig. 1A). PS metabolism is activated during adipogenesis (Horl et al., 2011). In the SAT of mice acutely exposed to cold, levels of PS and other phospholipids increase significantly (Xu et al., 2019). Furthermore, BAT undergoes dynamic phospholipid remodeling during thermogenesis, characterized by enrichment of PS species containing C18:2 chains, and coordinated changes in the expression of PS metabolic genes (Marcher et al., 2015). These findings suggest that PS exerts an active regulatory role in adipocyte metabolism beyond its traditional function as a structural membrane component. Indeed, a recent study suggests that dietary PS or PS-rich interventions could support metabolic health by activating fat breakdown and thermogenesis in adipose tissue (Zhou et al., 2024). Supplementing adipocytes and adipose tissue at the organismal level with PS increases lipolysis and thermogenesis. This is achieved by upregulating adipose triglyceride lipase and UCP1, a process that appears to be mediated likely by stabilizing PGC1α levels through direct PS binding and by stimulating the conventional cAMP-PKA signaling pathway (Zhou et al., 2024).

Cardiolipin, a distinct phospholipid species highly concentrated in the inner mitochondrial membrane, plays a central role in mitochondrial membrane architecture and electron transport chain function (Chicco and Sparagna, 2007, Paradies et al., 2014). In adipose, its synthesis is driven by the condensation of phosphatidylglycerol with an activated phosphatidyl group from CDP-diacylglycerol, with glycerol and fatty acyl residues derived from glycolysis and endogenous fatty acid synthesis (Jung et al., 2021, Schlein et al., 2021). Comprehensive lipidomic profiling has demonstrated that a specific increase in both the content and diversity of CL is a core signature of cold-induced metabolic adaptation in thermogenic adipose tissues (Lynes et al., 2018). The biosynthetic pathways of PG and CL are therefore crucial in both brown and beige fat. CL is not only enriched during thermogenic activation, but its synthesis and remodeling are also pivotal for supporting the mitochondrial structural integrity and bioenergetic capacity required for heat production (Lynes et al., 2018, Sustarsic et al., 2018). This is further supported by gain-of-function and loss-of-function experiments targeting the rate-limiting enzyme CL synthase (CRLS1), which highlights CL biosynthesis as a potential therapeutic target in metabolic disease (Sustarsic et al., 2018). In vitro studies suggest that CL may regulate UCP1 activity either by directly binding to the protein or by diminishing the binding of purine nucleotides, which are allosteric inhibitors of UCP1 (Fromme et al., 2018, Lee et al., 2015). However, the physiological relevance of these mechanisms in vivo remains unclear. Beyond synthesis, CL remodeling is equally important for maintaining mitochondrial integrity. Tafazzin (TAZ) transfers acyl chains to immature monolysocardiolipin to generate tetralinoleoyl cardiolipin (Schlame, 2013); the loss of *Taz* disrupts cristae structure and destabilizes the respiratory chain, ultimately impairing the thermogenic capacity of adipocytes (Johnson et al., 2020, McKenzie et al., 2006, Phoon et al., 2012). Recent studies have expanded CL biology beyond membrane structure, showing its association with various mitochondrial proteins. For instance, CL interacts with mitochondrial membrane-shaping proteins through conserved binding motifs to facilitate membrane remodeling (Thatavarthy et al., 2025). Furthermore, the exposure of CL on the outer mitochondrial membrane serves as an important signal for mitochondrial quality control, as shown by the fact that genetic deletion of *Crls1* impairs mitochondrial damage responses (Miao et al., 2023).

## PHOSPHOLIPID REMODELING

The dynamic remodeling of membrane phospholipids is crucial for maintaining cellular homeostasis and influencing various biological processes. The 2 fatty acyl chains that constitute phospholipids undergo constant remodeling through a pathway known as the Lands' cycle (Fig. 1B) (Lands, 1958, O'Donnell, 2022). This cycle alters the length or number of double bonds in the fatty acyl chains, which in turn modifies the biophysical properties of the phospholipid bilayer. The Lands' cycle consists of 2 key steps: deacylation and reacylation. In the deacylation step, phospholipase A2 (PLA2) hydrolyzes the fatty acyl chain at the sn-2 position of a phospholipid, generating a lysophospholipid (LysoPL) and a FFA. PLA2 is the central enzyme in this cycle and is found in the cytoplasm and membranes, though it can also be secreted (Khan and Ilies, 2023). Other phospholipases, such as phospholipase A1, phospholipase B, phospholipase C, and phospholipase D, do not directly participate in the acyl chain turnover characteristic of the Lands' cycle as they target different bonds or positions in phospholipids and mediate distinct reactions (Park et al., 2012). In the subsequent reacylation step, lysophospholipid acyltransferase (LPLAT) incorporates a specific fatty acid into the lysophospholipid at the sn-2 position, thereby completing the cycle (Harayama et al., 2014, Hashidate-Yoshida et al., 2015, Hishikawa et al., 2008). LPLAT is primarily localized in the ER but is also present in the membranes of mitochondria, the Golgi apparatus, and lipid droplets (Valentine et al., 2022) (Fig. 1B).

The PLA2 superfamily comprises 16 identified groups, categorized into 6 subfamilies based on their localization and substrate specificity: secreted PLA2, cytosolic PLA2 (cPLA2), Ca²⁺-independent PLA2, platelet-activating factor acetylhydrolase/lipoprotein-associated PLA2, lysosomal PLA2, and adipose-specific PLA2 (AdPLA). This diversity suggests their highly context-dependent functions. In adipose tissue, *AdPLA* (*Pla2g16*) has been the primary focus of research due to its high expression in adipocytes (Khan and Ilies, 2023), as discussed in a forthcoming section. However, the physiological contributions of other PLA2 subfamilies have been actively studied in non-adipose contexts. For instance, cPLA2 is ubiquitously expressed in nearly all brain cell types and is recognized for its regulation of synaptic plasticity (Sun et al., 2014). Platelet-activating factor acetylhydrolase is predominantly secreted by macrophages and has been established as a biomarker for cardiovascular disease (Mallat et al., 2010, Pantazi et al., 2022). Investigating these subfamilies within adipose tissue may be a worthwhile direction for future research.

**Fig. 2:**
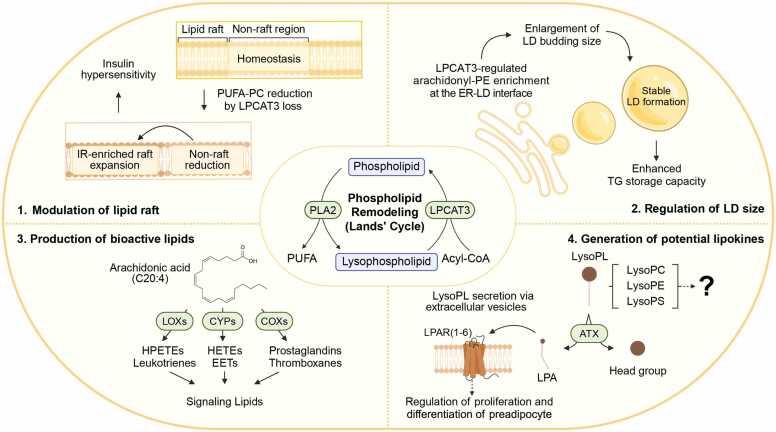
Versatile functions of phospholipid remodeling in adipocyte’s signaling and metabolism. (1) Lipid raft homeostasis. LPCAT3 deficiency results in expanded insulin receptor (IR)-rich raft domains and contracted nonraft domains as PUFA-PC decreases, thereby potentiating IR activation and insulin hypersensitivity. (2) Regulation of lipid droplet morphology and triglyceride storage capacity. LPCAT3 modulates the composition of phosphatidylethanolamine (PE), particularly arachidonoyl-PE, at the ER-lipid droplet (LD) interface, which is crucial for the formation and maturation of large lipid droplets. (3) Production of bioactive lipid mediator precursors. Arachidonic acid released by PLA2 is converted by cyclooxygenases (COXs), lipoxygenases (LOXs), and cytochrome P450s (CYPs) into diverse bioactive lipids, including prostaglandins, leukotrienes, and hydroxy-eicosatetraenoic acids (HETEs), which act as key signaling lipids. (4) Emergence of lysophospholipids (LysoPLs) with lipokine potential. PLA2 produces LysoPLs, including lysophosphatidylcholine (LysoPC), lysophosphatidylethanolamine (LysoPE), and lysophosphatidylserine (LysoPS), with diverse acyl chain compositions. The roles of these lysophospholipid species as lipokines are beginning to be appreciated. The best-understood example is lysophosphatidic acid (LPA), produced by autotaxin (ATX) from LysoPLs, which regulates preadipocyte proliferation and differentiation via G-protein-coupled receptor (GPCR) pathways.

The sn-2 position of phospholipids is often enriched with polyunsaturated fatty acids (PUFAs), including arachidonic acid (C20:4) and other long-chain PUFAs. These PUFAs released by PLA2 can be metabolized to form bioactive lipid mediators, including eicosanoids (Fig. 2). For example, arachidonic acid can be converted by cyclooxygenases (COXs) into prostaglandins and thromboxanes, by lipoxygenases (LOXs) into hydroperoxy-eicosatetraenoic acids (HPETEs), hydroxy-eicosatetraenoic acids (HETEs) and leukotrienes, and by cytochrome P450 into HETEs and epoxyeicosatrienoic acids (Harizi et al., 2008). These eicosanoids exhibit diverse biological functions, with some mediating proinflammatory responses while others possess anti-inflammatory or proresolving properties, and they have been implicated in cancer progression (Scher and Pillinger, 2009, Tsujii et al., 1998, Wang and Dubois, 2010). However, the physiological relevance of these proresolving lipid species remains debated, as they are only detectable at extremely low concentrations in tissue fluids (O'Donnell et al., 2023, Schebb et al., 2022).

Eicosanoids generated by PLA2 serve as important signaling molecules in adipose tissue lipid metabolism during both physiological and pathological conditions. Nutritional overload induces the expression and secretion of group IIA PLA2 (PLA2G2A), and pharmacological inhibition of this enzyme in rats has been shown to improve metabolic parameters, including insulin sensitivity and glucose tolerance (Iyer et al., 2012). Similarly, in vivo loss of *AdPLA/Pla2g16* in mice results in increased energy expenditure, which leads to a lean phenotype in mouse models of diet-induced and genetic obesity (Jaworski et al., 2009). The protective mechanisms suggested by both studies involve a reduction in the systemic release or adipose production of prostaglandin E2 (PGE2) (Iyer et al., 2012, Jaworski et al., 2009). PGE2 is the predominant prostaglandin produced in adipose tissue. Preadipocyte culture and adipose tissue explant experiments reveal that PGE2 biosynthesis is increased during adipogenesis and is more actively released from VAT than from SAT (Michaud et al., 2014). In mature adipocytes, PGE2 signals through the G-protein-coupled receptor EP3/PTGER3, activating the Gαi protein pathway and thereby suppressing cAMP-PKA-driven lipolysis as well as thermogenesis (Garcia-Alonso et al., 2016; Hu et al., 2016; Jaworski et al., 2009). However, it is important to note that the role of PGE2 and its associated pathways in adipose biology is complex, as the signaling outcomes can vary depending on which EP receptor (EP1-EP4/PTGER1-4) it binds to (Furuyashiki and Narumiya, 2011). This can lead to different effects on processes such as adipocyte differentiation and function. For instance, while PGE2-EP3 signaling suppresses lipolysis in white adipocytes, studies show that in brown adipocytes, PGE2 signaling through the EP2 and EP4 receptors positively regulates thermogenesis by promoting the transcriptional activation of UCP1 (Shamsi et al., 2020). Further studies using receptor-specific targeting approaches are needed to delineate context-dependent PGE2 functions in adipose tissue.

Adipocytes represent less than 50% of the total adipose depot; the remainder includes a diverse array of nonparenchymal cells such as adipocyte precursor cells, immune cells, nerve cells, and vascular cells (Corvera, 2021). Among them, immune cells can participate in the thermogenic remodeling of adipose tissue through PLA2 metabolism (Sato et al., 2020). Adipose M2 macrophages constitutively express secreted PLA2 (PLA2G2D), though its expression is suppressed during obesity. Mice with macrophage-specific *Pla2g2d* deficiency show reduced adipocyte browning and impaired adaptive thermogenesis. This is thought to be partly due to the enzyme's role in controlling omega-3 PUFA-driven signaling via the GPR120 receptor on adipocytes (Leiria and Tseng, 2020, Quesada-Lopez et al., 2016). Other studies show that inhibiting macrophage-specific de novo PC synthesis significantly reduces inflammation and insulin resistance in the WAT of *Lep*^*ob/ob*^ mice, an effect more pronounced than in liver or muscle (Petkevicius et al., 2019). These results support the idea that the phospholipid homeostasis of diverse cell types within the adipose niche collectively determines overall tissue function and systemic metabolic health.

LPLAT shapes membrane architecture by supplying PUFA-containing phospholipid (PUFA-PL) to cell membranes, which is key to providing membrane curvature and fluidity (Holthuis and Menon, 2014). Among the LPLAT family, lysophosphatidylcholine acyltransferase 3 (LPCAT3) is the predominant one in adipose tissue; however, its roles in adipose biology remain surprisingly undercharacterized and have only recently begun to be explored. Recent studies have focused on LPCAT3 in white adipose tissue lipid remodeling, drawing similar conclusions regarding LPCAT3 regulation of whole-body insulin sensitivity but through distinct mechanisms (He et al., 2023, Tol et al., 2025). Both groups used adipose-specific *Lpcat3* knockout mouse models to investigate the metabolic consequences of disrupting phospholipid remodeling in adipose tissue. He et al. (2023) focused on plasma membrane changes, finding that *Lpcat3* deficiency reduces PUFA-PC, which enhances lipid raft formation and, thereby boosting insulin receptor activation (Fig. 2). In contrast, Tol et al. showed that LPCAT3 incorporates arachidonic acid into PE at the ER-lipid droplet interface, generating a distinct arachidonyl-PE pool (Fig. 2). The formation of lipid droplets in adipocytes occurs through budding from the ER, and arachidonyl-PE enrichment at this interface is essential for forming large, stable lipid droplets (Iyer et al., 2012, Tol et al., 2025). Loss of *Lpcat3* disrupts this process, resulting in smaller, more unstable droplets susceptible to lipolysis, which causes lipodystrophy, ectopic fat deposition, and insulin resistance (Tol et al., 2025). Whether these proposed mechanisms represent mutually exclusive pathways or cell-type-specific processes within adipose tissue remains unresolved. These findings highlight how phospholipid remodeling regulates neutral lipid formation to maintain the optimal lipid storage capacity of adipose tissue. An exciting future direction would be to investigate more deeply the bidirectional crosstalk between phospholipid remodeling and neutral lipid metabolism—pathways that have long been studied in isolation—and how this interplay regulates optimal adipose lipid utilization.

**Fig. 3:**
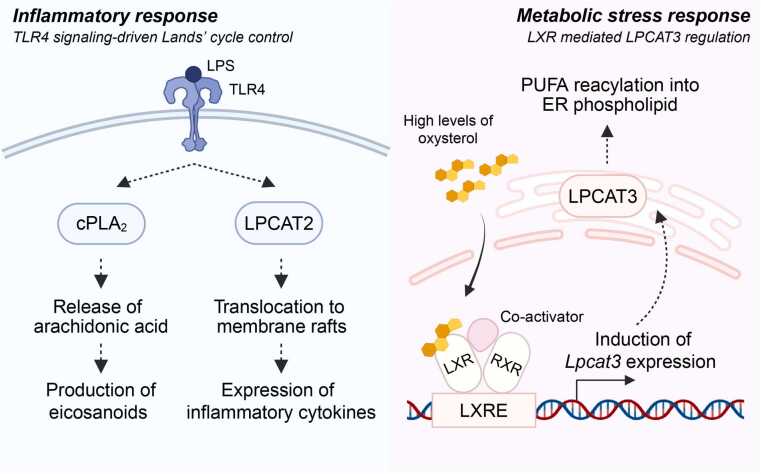
Regulation of the Lands' cycle under inflammatory and lipotoxic conditions. The Lands' cycle is regulated by multiple upstream factors. LPS binding to TLR4 activates cPLA2-dependent arachidonic acid release and promotes LPCAT2 translocation to membrane raft domains, thereby increasing eicosanoid levels that lead to inflammatory cytokine production. Under lipotoxic stress, excess intracellular cholesterol promotes oxysterol accumulation, which binds to and activates LXR in the nucleus. Ligand-bound LXR, already associated with RXR at the LXR response element (LXRE), activates transcription of target genes, including *Lpcat3*. This enhances PUFA reacylation into the ER membrane, thereby contributing to membrane remodeling.

The Lands' cycle generates potent lipid mediators and participates in signaling pathways that regulate diverse cellular functions. However, compared with other metabolic cycles, the regulation of the bidirectional deacylation/reacylation pathways within the Lands' cycle remains less understood, particularly in adipose tissue. Dynamic regulation of this cycle is exemplified in lipopolysaccharide (LPS)-stimulated macrophages, where LPS binding to Toll-like receptor 4 (TLR4) activates cPLA2 to stimulate eicosanoid production (Qi and Shelhamer, 2005) (Fig. 3). LPS-TLR4 signaling also modulates reacylation; for instance, LPS stimulation triggers the translocation of LPCAT2 to membrane raft domains where it interacts with TLR4 to facilitate p38 MAPK activation, driving inflammatory cytokine production (Abate et al., 2020). Furthermore, in response to lipotoxic stress, activation of liver X receptor (LXR) induces *Lpcat3* expression, which promotes reacylation and incorporation of unsaturated fatty acids into ER membrane phospholipids (Rong et al., 2013) (Fig. 3). Recent studies have confirmed the presence of this LXR-LPCAT3 axis in adipose tissue, establishing LXR as a key upstream regulator of the adipose Lands' cycle (Kleiboeker et al., 2024).

The ability of phospholipid remodeling pathways to generate PUFA-PL in membrane also links them to ferroptosis, a form of regulated cell death driven by iron-dependent lipid peroxidation (Jiang et al., 2021). The significance of this connection in adipose tissue is much less understood, but it suggests a new layer of control over adipocyte fate and function. Intriguingly, sublethal activation of ferroptotic pathways reduces lipid accumulation in adipocytes and attenuates diet-induced obesity in mice, while adipose tissue from humans with obesity exhibits diminished ferroptotic signatures, suggesting that impaired ferroptotic signaling may contribute to metabolic dysfunction (Wang et al., 2025). These findings point to ferroptosis modulation as a promising therapeutic avenue for obesity and its associated metabolic disorders.

Plasmalogen, a unique subclass of ether phospholipids characterized by a vinyl-ether bond at the sn-1 position, is highly abundant in human adipose tissue, with certain species rising in obesity, likely as an adaptive response to metabolic stress (Candi et al., 2018, Lange et al., 2021, Pietilainen et al., 2011). PLA2 catalyzes the hydrolysis of the sn-2 fatty acyl chain, generating lysoplasmalogens as key intermediates that link plasmalogen metabolism to lipid signaling and remodeling pathways, thereby influencing membrane architecture, inflammatory responses, and adipose physiology (Paul et al., 2019). Lysoplasmalogens can be further processed by additional phospholipases (phospholipase C, phospholipase D) and are ultimately catabolized by lysoplasmalogenase (Braverman and Moser, 2012). A recent study identified TMEM86A as an adipose-enriched lysoplasmalogenase (Cho et al., 2022). Adipocyte-specific deletion of *Tmem86a* increases lysoplasmalogen and plasmalogen levels, particularly LPC-P 18:0 and LPE-P 18:0 (Cho et al., 2022). These lysoplasmalogens stimulate cAMP-PKA signaling by inhibiting phosphodiesterase 3B, thereby enhancing mitochondrial oxidative metabolism and protecting against obesity.

Ceramide, a sphingolipid synthesized de novo from serine and palmitic acid, accumulates intracellularly when palmitic acid levels are in excess. This accumulation drives the secretion of proinflammatory adipokines (Chaurasia et al., 2020, Hamada et al., 2014). Ceramide modulates glucose and lipid metabolism in adipocytes through distinct mechanisms. First, it induces insulin resistance by blocking insulin-stimulated GLUT4 translocation. This occurs via activation of protein kinase C zeta, which inhibits Akt membrane recruitment, or through protein phosphatase 2A activation, which dephosphorylates Akt, thereby disrupting insulin signaling (Powell et al., 2003, Stratford et al., 2004, Stretton et al., 2010). Second, ceramide suppresses hormone-sensitive lipase activation in a protein phosphatase 2A-dependent manner, thereby attenuating lipolysis (Chaurasia et al., 2019). Mice with serine palmitoyltransferase (*Sptlc*) depletion, the rate-limiting enzyme of ceramide biosynthesis, exhibit adipocyte browning and protection from high-fat diet-induced obesity, supporting these findings (Chaurasia et al., 2016, Chaurasia et al., 2021). A recent study has identified formyl peptide receptor 2 as a membrane receptor for long-chain ceramides. Specifically, the binding of C16:0 ceramide to formyl peptide receptor 2 has been shown to inhibit adipocyte thermogenesis through Gi-coupled reduction of cAMP (Lin et al., 2025). Circulating ceramide levels have emerged as promising biomarkers for cardiometabolic diseases including cardiovascular disease and diabetes (Choi et al., 2021).

## LYSOPHOSPHOLIPIDS: EMERGING ADIPOKINES

Because adipose tissue handles large amounts of diverse lipid species, it is not surprising that it also generates and utilizes lipid-derived cytokines, called lipokines. Indeed, several lipokines are produced by adipose tissue and act in an autocrine and/or paracrine manner to regulate adipocyte function or systemic metabolism by interacting with other tissues (Cao et al., 2008, Gu et al., 2023). The most renowned lipokines are fatty acid derivatives, including branched fatty acid esters of hydroxy fatty acids (Yore et al., 2014), 12-hydroxyeicosapentaenoic acid (Leiria et al., 2019), and 12,13-dihydroxy-9Z-octadecenoic acid (12,13-diHOME) (Lynes et al., 2017), which have been extensively reviewed elsewhere (Tsuji and Tseng, 2023). Phospholipid remodeling pathways could contribute to lipokine production, as they generate both LysoPL, a class of bioactive lipids, and PUFA. While FFAs are released by lipolysis, LysoPLs are also released from adipocytes in various forms, including extracellular vesicles (Jakubec et al., 2020, Tan et al., 2020). These lipids can act in an autocrine/paracrine manner by binding to receptors on the adipocyte surface or perform endocrine roles by influencing other tissues. While the lipokine potential of PUFAs was discussed in the previous section, here we focus on covering LysoPLs.

The best-characterized LysoPLs, including lysophosphatidic acid (LPA) and sphingosine-1-phosphate (S1P), regulate cellular functions such as cell proliferation, survival, and Ca²⁺ homeostasis, and their dysregulation is associated with many diseases including cancer (Cuvillier et al., 1996, Nagahashi et al., 2018, Tigyi et al., 1994). LysoPLs exert signaling effects by binding to specific GPCRs (Blaho and Chun, 2018). For example, LPA binds to LPAR1-LPAR6, S1P binds to S1P1R-S1P5R (Yung et al., 2014). Though LysoPLs share similar overall structures within the same lipid class, how ligand specificity is determined—thought to depend on acyl chain length and degree of unsaturation—remains an active area of investigation (Liu et al., 2022, Troupiotis-Tsailaki et al., 2017).

LPA signaling represents the most extensively studied LysoPL-engaged signaling pathway in the context of adipose tissue (Fig. 2). Autotaxin (ATX; ENPP2), a secreted phosphodiesterase, converts LysoPLs such as LysoPC and LysoPS into bioactive LPA (Nakanaga et al., 2010). While both ATX and LPA levels are elevated in obesity (Ferry et al., 2003, Gesta et al., 2002), their functional impact is highly context-dependent, leading to seemingly contradictory experimental outcomes. For instance, whole-body *Enpp2*-deficient models or pharmacological inhibition of ATX/LPA signaling are protected from diet-induced obesity and show enhanced BAT activity, improved glucose tolerance, and muscle mitochondrial metabolism (D'Souza et al., 2018, Nishimura et al., 2014, Rancoule et al., 2013). In contrast, adipose-specific loss of *Enpp2* paradoxically increases adipose tissue mass by upregulating adipogenic markers and promoting local adiposity (Dusaulcy et al., 2011).

These discrepancies likely arise from the dual role of LPA in regulating the adipocyte life cycle through distinct molecular pathways. In early-stage preadipocytes, LPA-LPAR1 signaling activates the Ras-Raf-MEK-ERK pathway to drive cell proliferation and hyperplasia (Holmstrom et al., 2010). Conversely, in cells committing to differentiation, LPA acts as a potent anti-adipogenic factor by activating the Rho-ROCK pathway, which inhibits the master adipogenic transcriptional program (Li et al., 2011). This context-dependent response suggests that the ATX-LPA axis functions as a dynamic metabolic regulator, where the net effect on adiposity depends on the balance between progenitor expansion and the inhibition of mature adipocyte function. However, because these mechanisms have primarily been tested in vitro using 3T3-L1 and 3T3-F442A cells, further validation in vivo is required to fully understand their physiological relevance.

Whether other LysoPL species, which have recently gained considerable attention in many other biological contexts, contribute to adipose tissue growth and metabolism remains an open question (Liu et al., 2020, Trovato et al., 2023). Classic HPLC separation and phospholipidomic profiling studies have identified that LysoPC facilitates adipocyte glucose uptake and supports UCP1-mediated thermogenic respiration (Schweizer et al., 2019, Yea et al., 2009b). LysoPC generated as a downstream product of homocysteine metabolism can activate NLRP3 inflammasome signaling (Zhang et al., 2018). In vivo supplementation studies further support its potential anti-obesity effects (Han et al., 2021, Ma et al., 2021). LysoPS enhances glucose uptake in adipocytes, presumably through phosphatidylinositol-3-kinase signaling, and improves glycemic control in diabetic animal models (Yea et al., 2009a). While studies in other cells with active lipid metabolism, such as hepatocytes, show that LysoPE promotes lipid droplet formation and modifies genes involved in lipolysis (Yamamoto et al., 2022), its specific role in adipose tissue requires further investigation. Collectively, these findings highlight the diverse and expanding roles of LysoPL signaling at both cellular and systemic levels. Beyond acting as extracellular signaling molecules through GPCRs, LysoPLs and their metabolic intermediates (eg, LPA, FFAs hydrolyzed by lysophospholipase) may also function as intracellular signaling molecules or serve as substrates for other metabolic pathways (Nguyen et al., 2014, Wepy et al., 2019). Elucidating these potential dual roles represents an exciting direction for future research in the field.

## CONCLUSION AND PERSPECTIVE

While significant progress has been made in understanding the metabolism of phospholipids such as PC and CL, the roles of other phospholipid species in adipose tissue remain largely unexplored, representing a critical area for future research. A central theme emerging from recent work is the importance of phospholipid remodeling, particularly the Lands' cycle, as a key regulatory hub in adipose biology. Future studies could benefit from developing precise methods to measure the fate and flux of phospholipid species participating in this cycle. This will require the application of isotope tracing using nonradioactive stable lipid tracers coupled with high-resolution mass spectrometry to quantify the turnover rates of individual acyl chains analogous to approaches used for futile lipid cycling (Sharma et al., 2024, Wunderling et al., 2023), which would provide a more quantitative understanding of the dynamics of Lands’ cycle. The crosstalk between phospholipid remodeling and neutral lipid metabolism represents another promising research direction. As highlighted in this review, this interplay is crucial for processes ranging from insulin signaling to lipid droplet stability and thermogenic capacity. Investigating the regulation of phospholipid-driven protein-metabolite interactions is a rapidly growing research field (Hicks et al., 2023). Indeed, recent work reveals that LPCAT3-produced arachidonyl-PE binding to the electron transport chain component COX4I1 is required for optimal respiratory metabolism in brown adipocytes (Shimanaka et al., 2025).

Characterizing the heterogeneity of phospholipid metabolism across different adipose depots, as well as their distinct intratissue distributions, remains a critical future direction for the field. Lipidomic studies have revealed significant differences between SAT and VAT (Hou et al., 2020, Pradas et al., 2018). For instance, murine SAT is characterized by higher concentrations of ceramides compared with VAT. In overweight humans, SAT shows significant accumulation of PC, PI, and ether phospholipids enriched with highly unsaturated fatty acids. This accumulation may be attributed, at least in part, to the elevated expression of PEMT in SAT (Hou et al., 2020). However, the precise mechanistic links between these lipidomic signatures and depot-specific characteristics remain to be fully elucidated.

While earlier sections of this review cover several studies on the roles of nonparenchymal phospholipid metabolism in adipose tissue, this area remains largely unexplored. With advances in single-cell analysis technologies, lipidomic profiling is moving beyond bulk tissue analysis to the single-cell level (Sarkar and Ghosh, 2025). For example, spatial single-cell omics studies conducted in the liver have identified metabolic zonation-specific lipids (Tian et al., 2024). Applying these platforms to adipose tissue will be key to understanding cellular heterogeneity and its impact on tissue-level phospholipid homeostasis.

It should be noted that, although some clinical reports are available, most human data are still based on omics-level association studies. The mechanistic models of phospholipid metabolism discussed in this review have been primarily established using rodent models. Therefore, validating these rodent-derived mechanisms in human cohorts is a critical future direction to ensure clinical relevance, given species-specific differences in lipid metabolism, exemplified by lipid turnover rates (Arner et al., 2011). In this regard, phospholipid biosynthesis pathways are beginning to be examined through specific human genetic datasets—such as those involving PEMT and CRLS1 variants—underscoring the translational potential of targeting these pathways (Payne et al., 2014, Sharma et al., 2013).

As the field continues to uncover the multifaceted roles of phospholipid metabolism in adipose tissue, it becomes increasingly clear that these pathways are not merely structural housekeepers but active regulators of metabolic physiology and pathophysiology. Analyzing how phospholipid dynamics drive metabolic disease progression would be a vital next step. Specifically, future research should investigate the sequential phospholipid alterations during the development of obesity and their causal roles in promoting adipose tissue inflammation and systemic insulin resistance, as well as their contributions to other metabolic disorders such as lipodystrophy and cancer cachexia.

## Author Contributions

**Minju Kim:** Writing – review & editing, Writing – original draft, Project administration, Investigation, Conceptualization. **Hyunju Lee:** Writing – review & editing, Writing – original draft, Project administration, Investigation. **Joerg Heeren:** Writing – review & editing. **Junho Kim:** Writing – original draft, Writing – review & editing. **Su Myung Jung:** Writing – review & editing, Writing – original draft, Supervision, Conceptualization. **Gakyung Lee:** Writing – review & editing, Writing – original draft.

## Declaration of Competing Interests

The authors declare that they have no known competing financial interests or personal relationships that could have appeared to influence the work reported in this paper.

## References

[bib1] Abate W., Alrammah H., Kiernan M., Tonks A.J., Jackson S.K. (2020). Lysophosphatidylcholine acyltransferase 2 (LPCAT2) co-localises with TLR4 and regulates macrophage inflammatory gene expression in response to LPS. Sci. Rep..

[bib2] Ahmadian M., Duncan R.E., Jaworski K., Sarkadi-Nagy E., Sul H.S. (2007). Triacylglycerol metabolism in adipose tissue. Future Lipidol..

[bib3] Aitchison A.J., Arsenault D.J., Ridgway N.D. (2015). Nuclear-localized CTP:phosphocholine cytidylyltransferase alpha regulates phosphatidylcholine synthesis required for lipid droplet biogenesis. Mol. Biol. Cell.

[bib4] Arner P. (2005). Human fat cell lipolysis: biochemistry, regulation and clinical role. Best Pract. Res. Clin. Endocrinol. Metab..

[bib5] Arner P., Bernard S., Salehpour M., Possnert G., Liebl J., Steier P., Buchholz B.A., Eriksson M., Arner E., Hauner H. (2011). Dynamics of human adipose lipid turnover in health and metabolic disease. Nature.

[bib6] Bartelt A., Bruns O.T., Reimer R., Hohenberg H., Ittrich H., Peldschus K., Kaul M.G., Tromsdorf U.I., Weller H., Waurisch C. (2011). Brown adipose tissue activity controls triglyceride clearance. Nat. Med..

[bib7] Behrens J., Wang T., Kilian C., Worthmann A., Herman M.A., Heeren J., Adlung L., Scheja L. (2025). Single-nucleus mRNA-sequencing reveals dynamics of lipogenic and thermogenic adipocyte populations in murine brown adipose tissue in response to cold exposure. Mol. Metab..

[bib8] Blaho V.A., Chun J. (2018). 'Crystal' clear? Lysophospholipid receptor structure insights and controversies. Trends Pharmacol. Sci..

[bib9] Brasaemle D.L. (2007). Thematic review series: adipocyte biology. The perilipin family of structural lipid droplet proteins: stabilization of lipid droplets and control of lipolysis. J. Lipid Res..

[bib10] Brasaemle D.L., Dolios G., Shapiro L., Wang R. (2004). Proteomic analysis of proteins associated with lipid droplets of basal and lipolytically stimulated 3T3-L1 adipocytes. J. Biol. Chem..

[bib11] Braverman N.E., Moser A.B. (2012). Functions of plasmalogen lipids in health and disease. Biochim. Biophys. Acta.

[bib12] Candi E., Tesauro M., Cardillo C., Lena A.M., Schinzari F., Rodia G., Sica G., Gentileschi P., Rovella V., Annicchiarico-Petruzzelli M. (2018). Metabolic profiling of visceral adipose tissue from obese subjects with or without metabolic syndrome. Biochem. J..

[bib13] Cannon B., Nedergaard J. (2004). Brown adipose tissue: function and physiological significance. Physiol. Rev..

[bib14] Cao H., Gerhold K., Mayers J.R., Wiest M.M., Watkins S.M., Hotamisligil G.S. (2008). Identification of a lipokine, a lipid hormone linking adipose tissue to systemic metabolism. Cell.

[bib15] Cero C., Lea H.J., Zhu K.Y., Shamsi F., Tseng Y.H., Cypess A.M. (2021). beta3-Adrenergic receptors regulate human brown/beige adipocyte lipolysis and thermogenesis. JCI Insight.

[bib16] Chaurasia B., Kaddai V.A., Lancaster G.I., Henstridge D.C., Sriram S., Galam D.L., Gopalan V., Prakash K.N., Velan S.S., Bulchand S. (2016). Adipocyte ceramides regulate subcutaneous adipose browning, inflammation, and metabolism. Cell Metab..

[bib17] Chaurasia B., Talbot C.L., Summers S.A. (2020). Adipocyte ceramides-the Nexus of inflammation and metabolic disease. Front. Immunol..

[bib18] Chaurasia B., Tippetts T.S., Mayoral Monibas R., Liu J., Li Y., Wang L., Wilkerson J.L., Sweeney C.R., Pereira R.F., Sumida D.H. (2019). Targeting a ceramide double bond improves insulin resistance and hepatic steatosis. Science.

[bib19] Chaurasia B., Ying L., Talbot C.L., Maschek J.A., Cox J., Schuchman E.H., Hirabayashi Y., Holland W.L., Summers S.A. (2021). Ceramides are necessary and sufficient for diet-induced impairment of thermogenic adipocytes. Mol. Metab..

[bib20] Chicco A.J., Sparagna G.C. (2007). Role of cardiolipin alterations in mitochondrial dysfunction and disease. Am. J. Physiol. Cell Physiol..

[bib21] Cho Y.K., Lee S., Lee J., Doh J., Park J.H., Jung Y.S., Lee Y.H. (2023). Lipid remodeling of adipose tissue in metabolic health and disease. Exp. Mol. Med..

[bib22] Cho Y.K., Yoon Y.C., Im H., Son Y., Kim M., Saha A., Choi C., Lee J., Lee S., Kim J.H. (2022). Adipocyte lysoplasmalogenase TMEM86A regulates plasmalogen homeostasis and protein kinase A-dependent energy metabolism. Nat. Commun..

[bib23] Choi R.H., Tatum S.M., Symons J.D., Summers S.A., Holland W.L. (2021). Ceramides and other sphingolipids as drivers of cardiovascular disease. Nat. Rev. Cardiol..

[bib24] Cole L.K., Vance D.E. (2010). A role for Sp1 in transcriptional regulation of phosphatidylethanolamine N-methyltransferase in liver and 3T3-L1 adipocytes. J. Biol. Chem..

[bib25] Corvera S. (2021). Cellular heterogeneity in adipose tissues. Annu. Rev. Physiol..

[bib26] Cuvillier O., Pirianov G., Kleuser B., Vanek P.G., Coso O.A., Gutkind S., Spiegel S. (1996). Suppression of ceramide-mediated programmed cell death by sphingosine-1-phosphate. Nature.

[bib27] D'Souza K., Nzirorera C., Cowie A.M., Varghese G.P., Trivedi P., Eichmann T.O., Biswas D., Touaibia M., Morris A.J., Aidinis V. (2018). Autotaxin-LPA signaling contributes to obesity-induced insulin resistance in muscle and impairs mitochondrial metabolism. J. Lipid Res..

[bib28] DeLong C.J., Shen Y.J., Thomas M.J., Cui Z. (1999). Molecular distinction of phosphatidylcholine synthesis between the CDP-choline pathway and phosphatidylethanolamine methylation pathway. J. Biol. Chem..

[bib29] Donohoe C.L., Lysaght J., O'Sullivan J., Reynolds J.V. (2017). Emerging concepts linking obesity with the Hallmarks of cancer. Trends Endocrinol. Metab..

[bib30] Dusaulcy R., Rancoule C., Gres S., Wanecq E., Colom A., Guigne C., van Meeteren L.A., Moolenaar W.H., Valet P., Saulnier-Blache J.S. (2011). Adipose-specific disruption of autotaxin enhances nutritional fattening and reduces plasma lysophosphatidic acid. J. Lipid Res..

[bib31] Fagone P., Jackowski S. (2013). Phosphatidylcholine and the CDP-choline cycle. Biochim. Biophys. Acta.

[bib32] Ferry G., Tellier E., Try A., Gres S., Naime I., Simon M.F., Rodriguez M., Boucher J., Tack I., Gesta S. (2003). Autotaxin is released from adipocytes, catalyzes lysophosphatidic acid synthesis, and activates preadipocyte proliferation. Up-regulated expression with adipocyte differentiation and obesity. J. Biol. Chem..

[bib33] Flegal K.M., Kit B.K., Orpana H., Graubard B.I. (2013). Association of all-cause mortality with overweight and obesity using standard body mass index categories: a systematic review and meta-analysis. JAMA.

[bib34] Fromme T., Kleigrewe K., Dunkel A., Retzler A., Li Y., Maurer S., Fischer N., Diezko R., Kanzleiter T., Hirschberg V. (2018). Degradation of brown adipocyte purine nucleotides regulates uncoupling protein 1 activity. Mol. Metab..

[bib35] Fruhbeck G., Mendez-Gimenez L., Fernandez-Formoso J.A., Fernandez S., Rodriguez A. (2014). Regulation of adipocyte lipolysis. Nutr. Res. Rev..

[bib36] Furuyashiki T., Narumiya S. (2011). Stress responses: the contribution of prostaglandin E(2) and its receptors. Nat. Rev. Endocrinol..

[bib37] Gao X., van der Veen J.N., Hermansson M., Ordonez M., Gomez-Munoz A., Vance D.E., Jacobs R.L. (2015). Decreased lipogenesis in white adipose tissue contributes to the resistance to high fat diet-induced obesity in phosphatidylethanolamine N-methyltransferase-deficient mice. Biochim. Biophys. Acta.

[bib38] Garcia-Alonso V., Titos E., Alcaraz-Quiles J., Rius B., Lopategi A., Lopez-Vicario C., Jakobsson P.J., Delgado S., Lozano J., Claria J. (2016). Prostaglandin E2 exerts multiple regulatory actions on human obese adipose tissue remodeling, inflammation, adaptive thermogenesis and lipolysis. PLoS One.

[bib39] Gesta S., Simon M.F., Rey A., Sibrac D., Girard A., Lafontan M., Valet P., Saulnier-Blache J.S. (2002). Secretion of a lysophospholipase D activity by adipocytes: involvement in lysophosphatidic acid synthesis. J. Lipid Res..

[bib40] Gibellini F., Smith T.K. (2010). The Kennedy pathway--de novo synthesis of phosphatidylethanolamine and phosphatidylcholine. IUBMB Life.

[bib41] Gonzalez-Hurtado E., Lee J., Choi J., Wolfgang M.J. (2018). Fatty acid oxidation is required for active and quiescent brown adipose tissue maintenance and thermogenic programing. Mol. Metab..

[bib42] Grabner G.F., Xie H., Schweiger M., Zechner R. (2021). Lipolysis: cellular mechanisms for lipid mobilization from fat stores. Nat. Metab..

[bib43] Gu X., Wang L., Liu S., Shan T. (2023). Adipose tissue adipokines and lipokines: functions and regulatory mechanism in skeletal muscle development and homeostasis. Metabolism.

[bib44] Guilherme A., Rowland L.A., Wetoska N., Tsagkaraki E., Santos K.B., Bedard A.H., Henriques F., Kelly M., Munroe S., Pedersen D.J. (2023). Acetyl-CoA carboxylase 1 is a suppressor of the adipocyte thermogenic program. Cell Rep..

[bib45] Haemmerle G., Lass A., Zimmermann R., Gorkiewicz G., Meyer C., Rozman J., Heldmaier G., Maier R., Theussl C., Eder S. (2006). Defective lipolysis and altered energy metabolism in mice lacking adipose triglyceride lipase. Science.

[bib46] Hamada Y., Nagasaki H., Fujiya A., Seino Y., Shang Q.L., Suzuki T., Hashimoto H., Oiso Y. (2014). Involvement of de novo ceramide synthesis in pro-inflammatory adipokine secretion and adipocyte-macrophage interaction. J. Nutr. Biochem..

[bib47] Han A.R., Park H.R., Kim G.J., Kim B.R., Kim Y.R., Park H.H., Park J., Jin C.H., Kim J.M., Kwon S.J. (2021). 18:0 Lyso PC derived by bioactivity-based molecular networking from lentil mutant lines and its effects on high-fat diet-induced obese mice. Molecules.

[bib48] Harayama T., Eto M., Shindou H., Kita Y., Otsubo E., Hishikawa D., Ishii S., Sakimura K., Mishina M., Shimizu T. (2014). Lysophospholipid acyltransferases mediate phosphatidylcholine diversification to achieve the physical properties required in vivo. Cell Metab..

[bib49] Harizi H., Corcuff J.B., Gualde N. (2008). Arachidonic-acid-derived eicosanoids: Roles in biology and immunopathology. Trends Mol. Med..

[bib50] Hashidate-Yoshida T., Harayama T., Hishikawa D., Morimoto R., Hamano F., Tokuoka S.M., Eto M., Tamura-Nakano M., Yanobu-Takanashi R., Mukumoto Y. (2015). Fatty acid remodeling by LPCAT3 enriches arachidonate in phospholipid membranes and regulates triglyceride transport. Elife.

[bib51] He M., Li Z., Tung V.S.K., Pan M., Han X., Evgrafov O., Jiang X.C. (2023). Inhibiting phosphatidylcholine remodeling in adipose tissue increases insulin sensitivity. Diabetes.

[bib52] Hicks K.G., Cluntun A.A., Schubert H.L., Hackett S.R., Berg J.A., Leonard P.G., Ajalla Aleixo M.A., Zhou Y., Bott A.J., Salvatore S.R. (2023). Protein-metabolite interactomics of carbohydrate metabolism reveal regulation of lactate dehydrogenase. Science.

[bib53] Hishikawa D., Shindou H., Kobayashi S., Nakanishi H., Taguchi R., Shimizu T. (2008). Discovery of a lysophospholipid acyltransferase family essential for membrane asymmetry and diversity. Proc. Natl. Acad. Sci. U. S. A..

[bib54] Holmstrom T.E., Mattsson C.L., Wang Y., Iakovleva I., Petrovic N., Nedergaard J. (2010). Non-transactivational, dual pathways for LPA-induced Erk1/2 activation in primary cultures of brown pre-adipocytes. Exp. Cell Res..

[bib55] Holthuis J.C., Menon A.K. (2014). Lipid landscapes and pipelines in membrane homeostasis. Nature.

[bib56] Horl G., Wagner A., Cole L.K., Malli R., Reicher H., Kotzbeck P., Kofeler H., Hofler G., Frank S., Bogner-Strauss J.G. (2011). Sequential synthesis and methylation of phosphatidylethanolamine promote lipid droplet biosynthesis and stability in tissue culture and in vivo. J. Biol. Chem..

[bib57] Hou B., Zhao Y., He P., Xu C., Ma P., Lam S.M., Li B., Gil V., Shui G., Qiang G. (2020). Targeted lipidomics and transcriptomics profiling reveal the heterogeneity of visceral and subcutaneous white adipose tissue. Life Sci..

[bib58] Hu X., Cifarelli V., Sun S., Kuda O., Abumrad N.A., Su X. (2016). Major role of adipocyte prostaglandin E2 in lipolysis-induced macrophage recruitment. J. Lipid. Res..

[bib59] Huynh K., Barlow C.K., Jayawardana K.S., Weir J.M., Mellett N.A., Cinel M., Magliano D.J., Shaw J.E., Drew B.G., Meikle P.J. (2019). High-throughput plasma lipidomics: detailed mapping of the associations with cardiometabolic risk factors. Cell Chem. Biol..

[bib60] Iyer A., Lim J., Poudyal H., Reid R.C., Suen J.Y., Webster J., Prins J.B., Whitehead J.P., Fairlie D.P., Brown L. (2012). An inhibitor of phospholipase A2 group IIA modulates adipocyte signaling and protects against diet-induced metabolic syndrome in rats. Diabetes.

[bib61] Jakubec M., Maple-Grodem J., Akbari S., Nesse S., Halskau O., Mork-Jansson A.E. (2020). Plasma-derived exosome-like vesicles are enriched in lyso-phospholipids and pass the blood-brain barrier. PLoS One.

[bib62] Jaworski K., Ahmadian M., Duncan R.E., Sarkadi-Nagy E., Varady K.A., Hellerstein M.K., Lee H.Y., Samuel V.T., Shulman G.I., Kim K.H. (2009). AdPLA ablation increases lipolysis and prevents obesity induced by high-fat feeding or leptin deficiency. Nat. Med..

[bib63] Jiang X., Stockwell B.R., Conrad M. (2021). Ferroptosis: mechanisms, biology and role in disease. Nat. Rev. Mol. Cell Biol..

[bib64] Johnson J.M., Verkerke A.R.P., Maschek J.A., Ferrara P.J., Lin C.T., Kew K.A., Neufer P.D., Lodhi I.J., Cox J.E., Funai K. (2020). Alternative splicing of UCP1 by non-cell-autonomous action of PEMT. Mol. Metab..

[bib65] Jung S.M., Doxsey W.G., Le J., Haley J.A., Mazuecos L., Luciano A.K., Li H., Jang C., Guertin D.A. (2021). In vivo isotope tracing reveals the versatility of glucose as a brown adipose tissue substrate. Cell Rep..

[bib66] Kennedy E.P., Weiss S.B. (1956). The function of cytidine coenzymes in the biosynthesis of phospholipides. J. Biol. Chem..

[bib67] Khan S.A., Ilies M.A. (2023). The phospholipase A2 superfamily: structure, isozymes, catalysis, physiologic and pathologic roles. Int. J. Mol. Sci..

[bib68] Kim S., Moustaid-Moussa N. (2000). Secretory, endocrine and autocrine/paracrine function of the adipocyte. J. Nutr..

[bib69] Kleiboeker B., He A., Tan M., Lu D., Hu D., Liu X., Goodarzi P., Hsu F.F., Razani B., Semenkovich C.F. (2024). Adipose tissue peroxisomal lipid synthesis orchestrates obesity and insulin resistance through LXR-dependent lipogenesis. Mol. Metab..

[bib70] Korobkina E.D., Calejman C.M., Haley J.A., Kelly M.E., Li H., Gaughan M., Chen Q., Pepper H.L., Ahmad H., Boucher A. (2024). Brown fat ATP-citrate lyase links carbohydrate availability to thermogenesis and guards against metabolic stress. Nat. Metab..

[bib71] Krahmer N., Guo Y., Wilfling F., Hilger M., Lingrell S., Heger K., Newman H.W., Schmidt-Supprian M., Vance D.E., Mann M. (2011). Phosphatidylcholine synthesis for lipid droplet expansion is mediated by localized activation of CTP:phosphocholine cytidylyltransferase. Cell Metab..

[bib72] Lands W.E. (1958). Metabolism of glycerolipides; a comparison of lecithin and triglyceride synthesis. J. Biol. Chem..

[bib73] Lange M., Angelidou G., Ni Z., Criscuolo A., Schiller J., Bluher M., Fedorova M. (2021). AdipoAtlas: a reference lipidome for human white adipose tissue. Cell Rep. Med..

[bib74] Lass A., Zimmermann R., Haemmerle G., Riederer M., Schoiswohl G., Schweiger M., Kienesberger P., Strauss J.G., Gorkiewicz G., Zechner R. (2006). Adipose triglyceride lipase-mediated lipolysis of cellular fat stores is activated by CGI-58 and defective in Chanarin-Dorfman syndrome. Cell Metab..

[bib75] Lee S., Lim G., Kim S., Kim H., Roh Y.J., Kim W., Choi D.W., Jung S.M. (2023). Arteriovenous metabolomics to measure in vivo metabolite exchange in brown adipose tissue. J. Vis. Exp..

[bib76] Lee Y., Willers C., Kunji E.R., Crichton P.G. (2015). Uncoupling protein 1 binds one nucleotide per monomer and is stabilized by tightly bound cardiolipin. Proc. Natl. Acad. Sci. U. S. A..

[bib77] Leiria L.O., Tseng Y.H. (2020). Lipidomics of brown and white adipose tissue: Implications for energy metabolism. Biochim. Biophys. Acta Mol. Cell Biol. Lipids.

[bib78] Leiria L.O., Wang C.H., Lynes M.D., Yang K., Shamsi F., Sato M., Sugimoto S., Chen E.Y., Bussberg V., Narain N.R. (2019). 12-lipoxygenase regulates cold adaptation and glucose metabolism by producing the omega-3 lipid 12-HEPE from brown fat. Cell Metab..

[bib79] Li L., Tam L., Liu L., Jin T., Ng D.S. (2011). Wnt-signaling mediates the anti-adipogenic action of lysophosphatidic acid through cross talking with the Rho/Rho associated kinase (ROCK) pathway. Biochem. Cell Biol..

[bib80] Li Z., Agellon L.B., Allen T.M., Umeda M., Jewell L., Mason A., Vance D.E. (2006). The ratio of phosphatidylcholine to phosphatidylethanolamine influences membrane integrity and steatohepatitis. Cell Metab..

[bib81] Lin H., Ma C., Cai K., Guo L., Wang X., Lv L., Zhang C., Lin J., Zhang D., Ye C. (2025). Metabolic signaling of ceramides through the FPR2 receptor inhibits adipocyte thermogenesis. Science.

[bib82] Liu P., Zhu W., Chen C., Yan B., Zhu L., Chen X., Peng C. (2020). The mechanisms of lysophosphatidylcholine in the development of diseases. Life Sci..

[bib83] Liu S., Paknejad N., Zhu L., Kihara Y., Ray M., Chun J., Liu W., Hite R.K., Huang X.Y. (2022). Differential activation mechanisms of lipid GPCRs by lysophosphatidic acid and sphingosine 1-phosphate. Nat. Commun..

[bib84] Lodhi I.J., Yin L., Jensen-Urstad A.P., Funai K., Coleman T., Baird J.H., El Ramahi M.K., Razani B., Song H., Fu-Hsu F. (2012). Inhibiting adipose tissue lipogenesis reprograms thermogenesis and PPARgamma activation to decrease diet-induced obesity. Cell Metab..

[bib85] Lundgren P., Sharma P.V., Dohnalova L., Coleman K., Uhr G.T., Kircher S., Litichevskiy L., Bahnsen K., Descamps H.C., Demetriadou C. (2023). A subpopulation of lipogenic brown adipocytes drives thermogenic memory. Nat. Metab..

[bib86] Lynes M.D., Leiria L.O., Lundh M., Bartelt A., Shamsi F., Huang T.L., Takahashi H., Hirshman M.F., Schlein C., Lee A. (2017). The cold-induced lipokine 12,13-diHOME promotes fatty acid transport into brown adipose tissue. Nat. Med..

[bib87] Lynes M.D., Shamsi F., Sustarsic E.G., Leiria L.O., Wang C.H., Su S.C., Huang T.L., Gao F., Narain N.R., Chen E.Y. (2018). Cold-activated lipid dynamics in adipose tissue highlights a role for cardiolipin in thermogenic metabolism. Cell Rep..

[bib88] Ma Y., Du X., Zhao D., Tang K., Wang X., Guo S., Li X., Mei S., Sun N., Liu J. (2021). 18:0 Lyso PC, a natural product with potential PPAR-gamma agonistic activity, plays hypoglycemic effect with lower liver toxicity and cardiotoxicity in db/db mice. Biochem. Biophys. Res. Commun..

[bib89] Mallat Z., Lambeau G., Tedgui A. (2010). Lipoprotein-associated and secreted phospholipases A(2) in cardiovascular disease: Roles as biological effectors and biomarkers. Circulation.

[bib90] Marcher A.B., Loft A., Nielsen R., Vihervaara T., Madsen J.G., Sysi-Aho M., Ekroos K., Mandrup S. (2015). RNA-seq and mass-spectrometry-based lipidomics reveal extensive changes of glycerolipid pathways in brown adipose tissue in response to cold. Cell Rep..

[bib91] May F.J., Baer L.A., Lehnig A.C., So K., Chen E.Y., Gao F., Narain N.R., Gushchina L., Rose A., Doseff A.I. (2017). Lipidomic adaptations in white and brown adipose tissue in response to exercise demonstrate molecular species-specific remodeling. Cell Rep..

[bib92] McKenzie M., Lazarou M., Thorburn D.R., Ryan M.T. (2006). Mitochondrial respiratory chain supercomplexes are destabilized in Barth syndrome patients. J. Mol. Biol..

[bib93] Miao R., Jiang C., Chang W.Y., Zhang H., An J., Ho F., Chen P., Zhang H., Junqueira C., Amgalan D. (2023). Gasdermin D permeabilization of mitochondrial inner and outer membranes accelerates and enhances pyroptosis. Immunity.

[bib94] Michaud A., Lacroix-Pepin N., Pelletier M., Daris M., Biertho L., Fortier M.A., Tchernof A. (2014). Expression of genes related to prostaglandin synthesis or signaling in human subcutaneous and omental adipose tissue: depot differences and modulation by adipogenesis. Mediators Inflamm..

[bib95] Nagahashi M., Yamada A., Katsuta E., Aoyagi T., Huang W.C., Terracina K.P., Hait N.C., Allegood J.C., Tsuchida J., Yuza K. (2018). Targeting the SphK1/S1P/S1PR1 axis that links obesity, chronic inflammation, and breast cancer metastasis. Cancer Res..

[bib96] Nakanaga K., Hama K., Aoki J. (2010). Autotaxin--an LPA producing enzyme with diverse functions. J. Biochem..

[bib97] Nguyen L.N., Ma D., Shui G., Wong P., Cazenave-Gassiot A., Zhang X., Wenk M.R., Goh E.L., Silver D.L. (2014). Mfsd2a is a transporter for the essential omega-3 fatty acid docosahexaenoic acid. Nature.

[bib98] Nishimura S., Nagasaki M., Okudaira S., Aoki J., Ohmori T., Ohkawa R., Nakamura K., Igarashi K., Yamashita H., Eto K. (2014). ENPP2 contributes to adipose tissue expansion and insulin resistance in diet-induced obesity. Diabetes.

[bib99] O'Donnell V.B. (2022). New appreciation for an old pathway: the Lands Cycle moves into new arenas in health and disease. Biochem. Soc. Trans..

[bib100] O'Donnell V.B., Schebb N.H., Milne G.L., Murphy M.P., Thomas C.P., Steinhilber D., Gelhaus S.L., Kuhn H., Gelb M.H., Jakobsson P.J. (2023). Failure to apply standard limit-of-detection or limit-of-quantitation criteria to specialized pro-resolving mediator analysis incorrectly characterizes their presence in biological samples. Nat. Commun..

[bib101] Osuga J., Ishibashi S., Oka T., Yagyu H., Tozawa R., Fujimoto A., Shionoiri F., Yahagi N., Kraemer F.B., Tsutsumi O. (2000). Targeted disruption of hormone-sensitive lipase results in male sterility and adipocyte hypertrophy, but not in obesity. Proc. Natl. Acad. Sci. U. S. A..

[bib102] Pantazi D., Tellis C., Tselepis A.D. (2022). Oxidized phospholipids and lipoprotein-associated phospholipase A(2) (Lp-PLA(2)) in atherosclerotic cardiovascular disease: an update. Biofactors.

[bib103] Paradies G., Paradies V., De Benedictis V., Ruggiero F.M., Petrosillo G. (2014). Functional role of cardiolipin in mitochondrial bioenergetics. Biochim. Biophys. Acta.

[bib104] Park G., Haley J.A., Le J., Jung S.M., Fitzgibbons T.P., Korobkina E.D., Li H., Fluharty S.M., Chen Q., Spinelli J.B. (2023). Quantitative analysis of metabolic fluxes in brown fat and skeletal muscle during thermogenesis. Nat. Metab..

[bib105] Park J.B., Lee C.S., Jang J.H., Ghim J., Kim Y.J., You S., Hwang D., Suh P.G., Ryu S.H. (2012). Phospholipase signalling networks in cancer. Nat. Rev. Cancer.

[bib106] Paul S., Lancaster G.I., Meikle P.J. (2019). Plasmalogens: a potential therapeutic target for neurodegenerative and cardiometabolic disease. Prog. Lipid Res..

[bib107] Payne F., Lim K., Girousse A., Brown R.J., Kory N., Robbins A., Xue Y., Sleigh A., Cochran E., Adams C. (2014). Mutations disrupting the Kennedy phosphatidylcholine pathway in humans with congenital lipodystrophy and fatty liver disease. Proc. Natl. Acad. Sci. U. S. A..

[bib108] Petkevicius K., Virtue S., Bidault G., Jenkins B., Cubuk C., Morgantini C., Aouadi M., Dopazo J., Serlie M.J., Koulman A. (2019). Accelerated phosphatidylcholine turnover in macrophages promotes adipose tissue inflammation in obesity. Elife.

[bib109] Phoon C.K., Acehan D., Schlame M., Stokes D.L., Edelman-Novemsky I., Yu D., Xu Y., Viswanathan N., Ren M. (2012). Tafazzin knockdown in mice leads to a developmental cardiomyopathy with early diastolic dysfunction preceding myocardial noncompaction. J. Am. Heart Assoc..

[bib110] Pietilainen K.H., Rog T., Seppanen-Laakso T., Virtue S., Gopalacharyulu P., Tang J., Rodriguez-Cuenca S., Maciejewski A., Naukkarinen J., Ruskeepaa A.L. (2011). Association of lipidome remodeling in the adipocyte membrane with acquired obesity in humans. PLoS Biol..

[bib111] Powell D.J., Hajduch E., Kular G., Hundal H.S. (2003). Ceramide disables 3-phosphoinositide binding to the pleckstrin homology domain of protein kinase B (PKB)/Akt by a PKCzeta-dependent mechanism. Mol. Cell Biol..

[bib112] Pradas I., Huynh K., Cabre R., Ayala V., Meikle P.J., Jove M., Pamplona R. (2018). Lipidomics reveals a tissue-specific fingerprint. Front. Physiol..

[bib113] Qi H.Y., Shelhamer J.H. (2005). Toll-like receptor 4 signaling regulates cytosolic phospholipase A2 activation and lipid generation in lipopolysaccharide-stimulated macrophages. J. Biol. Chem..

[bib114] Quesada-Lopez T., Cereijo R., Turatsinze J.V., Planavila A., Cairo M., Gavalda-Navarro A., Peyrou M., Moure R., Iglesias R., Giralt M. (2016). The lipid sensor GPR120 promotes brown fat activation and FGF21 release from adipocytes. Nat. Commun..

[bib115] Rancoule C., Attane C., Gres S., Fournel A., Dusaulcy R., Bertrand C., Vinel C., Treguer K., Prentki M., Valet P. (2013). Lysophosphatidic acid impairs glucose homeostasis and inhibits insulin secretion in high-fat diet obese mice. Diabetologia.

[bib116] Roesler A., Kazak L. (2020). UCP1-independent thermogenesis. Biochem. J..

[bib117] Rong X., Albert C.J., Hong C., Duerr M.A., Chamberlain B.T., Tarling E.J., Ito A., Gao J., Wang B., Edwards P.A. (2013). LXRs regulate ER stress and inflammation through dynamic modulation of membrane phospholipid composition. Cell Metab..

[bib118] Sakers A., De Siqueira M.K., Seale P., Villanueva C.J. (2022). Adipose-tissue plasticity in health and disease. Cell.

[bib119] Sanchez-Gurmaches J., Tang Y., Jespersen N.Z., Wallace M., Martinez Calejman C., Gujja S., Li H., Edwards Y.J.K., Wolfrum C., Metallo C.M. (2018). Brown fat AKT2 is a cold-induced kinase that stimulates ChREBP-mediated de novo lipogenesis to optimize fuel storage and thermogenesis. Cell Metab..

[bib120] Sarkar S., Ghosh R. (2025). Unravelling lipid heterogeneity: advances in single-cell lipidomics in cellular metabolism and disease. BBA Adv..

[bib121] Sato H., Taketomi Y., Miki Y., Murase R., Yamamoto K., Murakami M. (2020). Secreted phospholipase PLA2G2D contributes to metabolic health by mobilizing omega3 polyunsaturated fatty acids in WAT. Cell Rep..

[bib122] Schebb N.H., Kuhn H., Kahnt A.S., Rund K.M., O'Donnell V.B., Flamand N., Peters-Golden M., Jakobsson P.J., Weylandt K.H., Rohwer N. (2022). Formation, signaling and occurrence of specialized pro-resolving lipid mediators-what is the evidence so far?. Front. Pharmacol..

[bib123] Scher J.U., Pillinger M.H. (2009). The anti-inflammatory effects of prostaglandins. J. Investig. Med..

[bib124] Schlame M. (2013). Cardiolipin remodeling and the function of tafazzin. Biochim. Biophys. Acta.

[bib125] Schlame M., Greenberg M.L. (2017). Biosynthesis, remodeling and turnover of mitochondrial cardiolipin. Biochim Biophys Acta Mol Cell Biol Lipids.

[bib126] Schlein C., Fischer A.W., Sass F., Worthmann A., Todter K., Jaeckstein M.Y., Behrens J., Lynes M.D., Kiebish M.A., Narain N.R. (2021). Endogenous fatty acid synthesis drives brown adipose tissue involution. Cell Rep..

[bib127] Schweizer S., Liebisch G., Oeckl J., Hoering M., Seeliger C., Schiebel C., Klingenspor M., Ecker J. (2019). The lipidome of primary murine white, brite, and brown adipocytes-Impact of beta-adrenergic stimulation. PLoS Biol..

[bib128] Shamsi F., Xue R., Huang T.L., Lundh M., Liu Y., Leiria L.O., Lynes M.D., Kempf E., Wang C.H., Sugimoto S. (2020). FGF6 and FGF9 regulate UCP1 expression independent of brown adipogenesis. Nat. Commun..

[bib129] Sharma A.K., Khandelwal R., Wolfrum C. (2024). Futile cycles: emerging utility from apparent futility. Cell Metab..

[bib130] Sharma N.K., Langberg K.A., Mondal A.K., Das S.K. (2013). Phospholipid biosynthesis genes and susceptibility to obesity: Analysis of expression and polymorphisms. PLoS One.

[bib131] Shimanaka, Y., Tol, M.J., Rocha-Roa, C., Jellinek, M.J., Cui, L., Bender, A., Bedard, A.H., Milner, M.G., Melillo, B., Shoucri, B.M., et al. (2025). A cold-inducible phospholipid-protein interaction in brown fat mitochondria optimizes thermogenic capacity. bioRxiv [Preprint], PMID: 40463239. 10.1101/2025.05.15.654206.

[bib132] Sidossis L., Kajimura S. (2015). Brown and beige fat in humans: thermogenic adipocytes that control energy and glucose homeostasis. J. Clin. Invest..

[bib133] Simcox J., Geoghegan G., Maschek J.A., Bensard C.L., Pasquali M., Miao R., Lee S., Jiang L., Huck I., Kershaw E.E. (2017). Global analysis of plasma lipids identifies liver-derived acylcarnitines as a fuel source for brown fat thermogenesis. Cell Metab..

[bib134] Spector A.A., Yorek M.A. (1985). Membrane lipid composition and cellular function. J. Lipid Res..

[bib135] Stratford S., Hoehn K.L., Liu F., Summers S.A. (2004). Regulation of insulin action by ceramide: dual mechanisms linking ceramide accumulation to the inhibition of Akt/protein kinase B. J. Biol. Chem..

[bib136] Stretton C., Evans A., Hundal H.S. (2010). Cellular depletion of atypical PKClambda is associated with enhanced insulin sensitivity and glucose uptake in L6 rat skeletal muscle cells. Am. J. Physiol. Endocrinol. Metab..

[bib137] Sun G.Y., Chuang D.Y., Zong Y., Jiang J., Lee J.C., Gu Z., Simonyi A. (2014). Role of cytosolic phospholipase A2 in oxidative and inflammatory signaling pathways in different cell types in the central nervous system. Mol. Neurobiol..

[bib138] Sustarsic E.G., Ma T., Lynes M.D., Larsen M., Karavaeva I., Havelund J.F., Nielsen C.H., Jedrychowski M.P., Moreno-Torres M., Lundh M. (2018). Cardiolipin synthesis in brown and beige fat mitochondria is essential for systemic energy homeostasis. Cell Metab..

[bib139] Tan S.T., Ramesh T., Toh X.R., Nguyen L.N. (2020). Emerging roles of lysophospholipids in health and disease. Prog. Lipid Res..

[bib140] Tansey J.T., Sztalryd C., Gruia-Gray J., Roush D.L., Zee J.V., Gavrilova O., Reitman M.L., Deng C.X., Li C., Kimmel A.R. (2001). Perilipin ablation results in a lean mouse with aberrant adipocyte lipolysis, enhanced leptin production, and resistance to diet-induced obesity. Proc. Natl. Acad. Sci. U. S. A..

[bib141] Thatavarthy S., Abriata L.A., Meireles F.T.P., Zuccaro K.E., Gargey Iragavarapu A., Sullivan G.M., Moss F.R., Frost A., Dal Peraro M., Aydin H. (2025). Cardiolipin dynamics promote membrane remodeling by mitochondrial OPA1. Nat. Commun..

[bib142] Tian H., Rajbhandari P., Tarolli J., Decker A.M., Neelakantan T.V., Angerer T., Zandkarimi F., Remotti H., Frache G., Winograd N. (2024). Multimodal mass spectrometry imaging identifies cell-type-specific metabolic and lipidomic variation in the mammalian liver. Dev. Cell.

[bib143] Tigyi G., Dyer D.L., Miledi R. (1994). Lysophosphatidic acid possesses dual action in cell proliferation. Proc. Natl. Acad. Sci. U. S. A..

[bib144] Tol M.J., Shimanaka Y., Bedard A.H., Sapia J., Cui L., Colaco-Gaspar M., Hofer P., Ferrari A., Qian K., Kennelly J.P. (2025). Dietary control of peripheral adipose storage capacity through membrane lipid remodelling. Nat. Metab..

[bib145] Trayhurn P., Beattie J.H. (2001). Physiological role of adipose tissue: white adipose tissue as an endocrine and secretory organ. Proc. Nutr. Soc..

[bib146] Troupiotis-Tsailaki A., Zachmann J., Gonzalez-Gil I., Gonzalez A., Ortega-Gutierrez S., Lopez-Rodriguez M.L., Pardo L., Govaerts C. (2017). Ligand chain length drives activation of lipid G protein-coupled receptors. Sci. Rep..

[bib147] Trovato F.M., Zia R., Artru F., Mujib S., Jerome E., Cavazza A., Coen M., Wilson I., Holmes E., Morgan P. (2023). Lysophosphatidylcholines modulate immunoregulatory checkpoints in peripheral monocytes and are associated with mortality in people with acute liver failure. J. Hepatol..

[bib148] Tsuji T., Tseng Y.H. (2023). Adipose tissue-derived lipokines in metabolism. Curr. Opin. Genet. Dev..

[bib149] Tsujii M., Kawano S., Tsuji S., Sawaoka H., Hori M., DuBois R.N. (1998). Cyclooxygenase regulates angiogenesis induced by colon cancer cells. Cell.

[bib150] Valentine W.J., Yanagida K., Kawana H., Kono N., Noda N.N., Aoki J., Shindou H. (2022). Update and nomenclature proposal for mammalian lysophospholipid acyltransferases, which create membrane phospholipid diversity. J. Biol. Chem..

[bib151] van Meer G., Voelker D.R., Feigenson G.W. (2008). Membrane lipids: where they are and how they behave. Nat. Rev. Mol. Cell Biol..

[bib152] Vance J.E. (2015). Phospholipid synthesis and transport in mammalian cells. Traffic.

[bib153] Vazquez-Vela M.E., Torres N., Tovar A.R. (2008). White adipose tissue as endocrine organ and its role in obesity. Arch. Med. Res..

[bib154] Wang D., Dubois R.N. (2010). Eicosanoids and cancer. Nat. Rev. Cancer.

[bib155] Wang X., Wu Q., Zhong M., Chen Y., Wang Y., Li X., Zhao W., Ge C., Wang X., Yu Y. (2025). Adipocyte-derived ferroptotic signaling mitigates obesity. Cell Metab..

[bib156] Wang Z., Ning T., Song A., Rutter J., Wang Q.A., Jiang L. (2020). Chronic cold exposure enhances glucose oxidation in brown adipose tissue. EMBO Rep..

[bib157] Wepy J.A., Galligan J.J., Kingsley P.J., Xu S., Goodman M.C., Tallman K.A., Rouzer C.A., Marnett L.J. (2019). Lysophospholipases cooperate to mediate lipid homeostasis and lysophospholipid signaling. J. Lipid Res..

[bib158] Wunderling K., Zurkovic J., Zink F., Kuerschner L., Thiele C. (2023). Triglyceride cycling enables modification of stored fatty acids. Nat. Metab..

[bib159] Wymann M.P., Schneiter R. (2008). Lipid signalling in disease. Nat. Rev. Mol. Cell Biol..

[bib160] Xu Z., You W., Zhou Y., Chen W., Wang Y., Shan T. (2019). Cold-induced lipid dynamics and transcriptional programs in white adipose tissue. BMC Biol..

[bib161] Yamamoto Y., Sakurai T., Chen Z., Inoue N., Chiba H., Hui S.P. (2022). Lysophosphatidylethanolamine Affects lipid accumulation and metabolism in a human liver-derived cell line. Nutrients.

[bib162] Yea K., Kim J., Lim S., Kwon T., Park H.S., Park K.S., Suh P.G., Ryu S.H. (2009). Lysophosphatidylserine regulates blood glucose by enhancing glucose transport in myotubes and adipocytes. Biochem. Biophys. Res. Commun..

[bib163] Yea K., Kim J., Yoon J.H., Kwon T., Kim J.H., Lee B.D., Lee H.J., Lee S.J., Kim J.I., Lee T.G. (2009). Lysophosphatidylcholine activates adipocyte glucose uptake and lowers blood glucose levels in murine models of diabetes. J. Biol. Chem..

[bib164] Yoneshiro T., Wang Q., Tajima K., Matsushita M., Maki H., Igarashi K., Dai Z., White P.J., McGarrah R.W., Ilkayeva O.R. (2019). BCAA catabolism in brown fat controls energy homeostasis through SLC25A44. Nature.

[bib165] Yore M.M., Syed I., Moraes-Vieira P.M., Zhang T., Herman M.A., Homan E.A., Patel R.T., Lee J., Chen S., Peroni O.D. (2014). Discovery of a class of endogenous mammalian lipids with anti-diabetic and anti-inflammatory effects. Cell.

[bib166] Yung Y.C., Stoddard N.C., Chun J. (2014). LPA receptor signaling: pharmacology, physiology, and pathophysiology. J. Lipid Res..

[bib167] Zhang S.Y., Dong Y.Q., Wang P., Zhang X., Yan Y., Sun L., Liu B., Zhang D., Zhang H., Liu H. (2018). Adipocyte-derived lysophosphatidylcholine activates adipocyte and adipose tissue macrophage nod-like receptor protein 3 inflammasomes mediating homocysteine-induced insulin resistance. EBioMedicine.

[bib168] Zhou Y., Ling D., Wang L., Xu Z., You W., Chen W., Nong Q., Valencak T.G., Shan T. (2024). Dietary "Beigeing" fat contains more phosphatidylserine and enhances mitochondrial function while counteracting obesity. Research (Wash. D. C.).

[bib169] Zimmermann R., Strauss J.G., Haemmerle G., Schoiswohl G., Birner-Gruenberger R., Riederer M., Lass A., Neuberger G., Eisenhaber F., Hermetter A. (2004). Fat mobilization in adipose tissue is promoted by adipose triglyceride lipase. Science.

